# Potential of periosteal cells in bone and cartilage regeneration: a systematic review

**DOI:** 10.3389/fbioe.2023.1292483

**Published:** 2023-11-09

**Authors:** Rongkai Cao, Beibei Chen, Qianru Li, Piaopiao Qiu, Xiaojie Liang, Yujie Cao

**Affiliations:** ^1^ Stomatological Hospital and Dental School of Tongji University, Shanghai Engineering Research Center of Tooth Restoration and Regeneration, Shanghai, China; ^2^ Department of Stomatology, The First Affiliated Hospital of Fujian Medical University, Fuzhou, China; ^3^ Department of Stomatology, People’s Hospital of Xiangyun Affiliated to Dali University, Dali, China

**Keywords:** periosteal cells, bone marrow stromal cells, bone regeneration, cartilage regeneration, tissue engineering

## Abstract

**Introduction:** The unavailability of adequate human primary cells presents multiple challenges in terms of bone and cartilage regeneration and disease modeling experiments *in vitro*. Periosteal cells (PCs), which represent promising skeletal stem cell sources, could be a promising strategy in tissue engineering. The present study aimed to summarize the characteristics of PCs to investigate the efficacy of these cells in bone and cartilage regeneration in different models, paying special attention to the comparison of bone marrow stromal cells (BMSCs).

**Methods:** A comprehensive literature search was conducted in Embase, PubMed/MEDLINE, Web of Science, and Scopus for articles published in English until April 2023. Only original researches in which PCs were employed for bone or cartilage regeneration experiments were included.

**Results:** A total of 9140 references were retrieved. After screening the results, 36 publications were considered to be eligible for inclusion in the present literature review. Overall, PCs demonstrated beneficial bone and cartilage regenerative efficacy compared to the bare scaffold since almost all included studies reported positive results. The 9 studies assessing the differences in bone formation capacity between PCs and BMSCs indicated that PCs exhibited stronger *in vivo* osteogenic differentiation capabilities compared to BMSCs, while the other study demonstrated stronger chondrogenic potential of BMSCs.

**Discussion:** PCs demonstrated beneficial to bone regenerative efficacy compared to the bare scaffold with a low risk of most studies included. However, the cartilage formation capacity of BMSCs still needs to be investigated due to the limited research available and the certain risk of bias. PCs exhibited higher osteogenic capabilities compared to BMSCs in combination with various scaffolds *in vivo* with good evidence. Further researches are needed to elucidate the comparative benefits of cartilage regeneration.

**Systematic Review Registration:** https://www.crd.york.ac.uk/prospero/display_record.php?ID=CRD42023411522, CRD42023411522.

## Introduction

Over the past decades, therapeutic options capable of repairing and reconstructing bone and cartilage defects have attracted a great deal of scientific and public attention (Grayson et al., 2015; Tamaddon et al., 2018). Normally, small defects can be effectively repaired because of the regenerative potential of bone and cartilage tissues. However, large ones due to multiple diseases remain a great challenge in clinical scenarios (Atala et al., 2012; Su et al., 2018). In addition, the morbidity of musculoskeletal disorders including fractures, osteoporosis, and rheumatic diseases is increasing rapidly due to the increased life expectancy (Roseti et al., 2017). Recently, the conventional approach to cure complex large bone defects includes transplantation of allogenous or autogenous bone grafts harvesting from the radius, fibula, iliac crest, and scapula or the application of substitutes to restore bone integrity (Toros and Ozaksar, 2021). Nevertheless, the inherent shortcomings of this method, such as donor-site morbidity and insufficient autogenous resources, significantly encourage researchers and clinicians to explore alternative treatment options (Dimitriou et al., 2011). Surgical options to manage damaged cartilage include arthroscopic debridement, osteochondral allograft, osteochondral autografts, and, in the presence of osteoarthritis, joint replacement (Goldberg et al., 2017). Bone marrow stimulation techniques, such as micro-fracture, are the most frequently used method in clinical practice for treating small symptomatic lesions of the articular cartilage (Steinwachs et al., 2008). However, the resulting tissue has shown to be a mixed fibrocartilage tissue with varying amounts of type II collagen and inferior to native hyaline cartilage (Goldberg et al., 2017). In this context, tissue engineering based on stem cells and scaffolds has emerged as a potential alternative method for the replacement of defective or malfunctioning tissues. This approach eliminates the inherent limitations of traditional transplantation of bone grafts and provides biological tissue substitutes in various conditions. Through recapitulating critical features of development or tissue repair, stem cell-based tissue engineering can improve tissue formation *in vitro* or promote tissue regeneration *in vivo* for the replacement of damaged ones (Charwat et al., 2008; Jukes et al., 2010).

Stem cells are defined as a population of undifferentiated cells with the potential to extensively proliferate from a single cell to different types of cells and tissues (Kolios and Moodley, 2013). Because of the unique ability including self-renew and multidirectional differentiation, tissues that can be engineered using these cells comprise a diverse range from skeletal tissues to epithelial surfaces, which present unprecedented applications. Stem cells are indispensable for the practical use of tissue engineering approaches, and the acquisition of stem cells is important. Among various sources of stem cells used for bone and cartilage regeneration, the bone marrow compartment has been demonstrated to represent a reliable tissue resource to harvest stem cells with convincing evidence of differentiation capacity both *in vitro* and *in vivo* (Li et al., 2009; Arthur and Gronthos, 2020). In addition, the periosteum is another essential source of mesenchymal stem cells (MSCs) for cartilage and bone regeneration in addition to the bone marrow compartment, which was originally identified as a reliable resource to harvest MSCs (Bolander et al., 2017; Mendes et al., 2018).

As an essential component covering the outer surface of bone, the periosteum is of great significance in bone physiology during remodeling, development, and growth (Maia Ferreira Alencar et al., 2020). Its structure is heterogeneous, consisting of the following two layers: the outer fibrous layer with fibroblasts, and the inner cambium layer, which contains osteoprogenitor cells, osteoblasts, and pre-osteoblasts that influence bone formation. Activated periosteum produces cartilage and bone, and is colonized by osteoclasts (Hutmacher and Sittinger, 2003). As a primary source of MSCs, PCs have gained a lot of scientific attention for regenerative approaches. The capacity of PCs to develop into bone and cartilage has been demonstrated in several studies (Miyamoto et al., 2004; Chen et al., 2012). In addition, with the help of continuous development of tools and techniques, specific role and regulation of PCs can be investigated more deeply since the challenge of isolating PCs has been overcome.

Previously published systematic reviews have proved the efficacy of BMSCs for bone and cartilage regeneration (Sun et al., 2016; Zhu et al., 2023). However, the role of PCs in tissue engineering remains unclear. Accordingly, it is necessary to summarize the current evidence in terms of the application of PCs in bone and cartilage regeneration. Therefore, the aim of this study is to conduct a systemic review to assess the osteogenic and chondrogenic capacities of PCs. In addition, this review also elucidates the limitations of existing research, paying special attention to the comparison of bone marrow stromal cells (BMSCs). To our knowledge, this is one of the first reviews that summarizes the potential role of PCs in both bone and cartilage regeneration.

## Materials and methods

The present systematic review was registered at PROSPERO under number CRD42023411522 and performed in accordance with the Preferred Reporting Items for Systematic Reviews and Meta-analyses (PRISMA) guidelines (Moher et al., 2015). As this study did not involve human or animal subjects, ethics approval was not required.

The guiding question for this systematic review was formulated according to the PICO format; (P) indicates the participants, (I) means the intervention, (C) represents the comparison, and (O) is for the outcome (Schardt et al., 2007). Does the use of PCs (I) improve the rate of bone and cartilage regeneration (O), compared to formation ability using other types of cells (C) in various animal models (P)?

### Search strategy

A systematic literature search was performed in Embase, PubMed/MEDLINE, Web of Science, and Scopus as sources for literature published up to April 2023 to identify suitable publications. These four databases were selected since they are the largest pharmaceutical and biomedical databases, which would be unlikely to lessen the number of articles. Defense Technical Information Center was used to search gray literature. The search strategy was shown in Table 1. Three components were included: bone regeneration and/or cartilage regeneration, PCs and tissue engineering. In addition, the electronic search of the databases was complemented by a manual search in reference lists of chosen articles to improve completeness.

**TABLE 1:** Electronic databases used and search strategies.

Database	Search strategy
PubMed	((periosteal cell)[All Fields] OR (periosteum cell)[All Fields] OR (periosteum derived cell)[All Fields])) AND (bone[All Fields] OR cartilage[All Fields]) AND (regeneration[All Fields] OR repair[All Fields] OR formation[All Fields] OR reconstruction[All Fields] OR healing[All Fields] OR engineering[All Fields] OR augmentation[All Fields]) AND ((stem cell)[All Fields] OR (cell culture)[All Fields] OR (cell transplantation)[All Fields] OR (cell engineering)[All Fields] OR (tissue engineering)[All Fields] OR (tissue culture)[All Fields] OR (tissue engineered)[All Fields])
Scopus	(TITLE-ABS-KEY(periosteal cell) OR TITLE-ABS-KEY(periosteum cell) OR TITLE-ABS-KEY(periosteum derived cell)) AND (TITLE-ABS-KEY(bone) OR TITLE-ABS-KEY(cartilage)) AND (TITLE-ABS-KEY(regeneration) OR TITLE-ABS-KEY(repair) OR TITLE-ABS-KEY(formation) OR TITLE-ABS-KEY(reconstruction) OR TITLE-ABS-KEY(healing) OR TITLE-ABS-KEY(engineering) OR TITLE-ABS-KEY(augmentation)) AND (TITLE-ABS-KEY(stem cell) OR TITLE-ABS-KEY(cell culture) OR TITLE-ABS-KEY(cell transplantation) OR TITLE-ABS-KEY(cell engineering) OR TITLE-ABS-KEY(tissue engineering) OR TITLE-ABS-KEY(tissue culture) OR TITLE-ABS-KEY(tissue engineered))
Web of science	(((TS=((periosteal cell OR periosteum cell OR periosteum derived cell))) AND TS=((bone OR cartilage))) AND TS=((regeneration OR repair OR formation OR reconstruction OR healing OR engineering OR augmentation))) AND TS=((stem cell OR cell culture OR cell transplantation OR cell engineering OR tissue engineering OR tissue culture OR tissue engineered))
Embase	(‘periosteal cell’ OR ((‘periosteal’/exp OR periosteal) AND (‘cell’/exp OR cell)) OR ‘periosteum cell’ OR ((‘periosteum’/exp OR periosteum) AND (‘cell’/exp OR cell)) OR ‘periosteum derived cell’/exp OR ‘periosteum derived cell’ OR ((‘periosteum’/exp OR periosteum) AND derived AND (‘cell’/exp OR cell))) AND (‘bone’/exp OR bone OR ‘cartilage’/exp OR cartilage) AND (‘regeneration’/exp OR regeneration OR ‘repair’/exp OR repair OR formation OR ‘reconstruction’/exp OR reconstruction OR ‘healing’/exp OR healing OR ‘engineering’/exp OR engineering OR ‘augmentation’/exp OR augmentation) AND (‘stem cell’/exp OR ‘stem cell' OR ((‘stem’/exp OR stem) AND (‘cell’/exp OR cell)) OR ‘cell culture’/exp OR ‘cell culture’ OR ((‘cell’/exp OR cell) AND (‘culture’/exp OR culture)) OR ‘cell transplantation’/exp OR ‘cell transplantation’ OR ((‘cell’/exp OR cell) AND (‘transplantation’/exp OR transplantation)) OR ‘cell engineering’/exp OR ‘cell engineering’ OR ((‘cell’/exp OR cell) AND (‘engineering’/exp OR engineering)) OR ‘tissue engineering’/exp OR ‘tissue engineering’ OR ((‘tissue’/exp OR tissue) AND (‘engineering’/exp OR engineering)) OR ‘tissue culture’/exp OR ‘tissue culture’ OR ((‘tissue’/exp OR tissue) AND (‘culture’/exp OR culture)) OR ‘tissue engineered’ OR ((‘tissue’/exp OR tissue) AND engineered))

### Eligibility criteria

Publications that fulfilled the following inclusion criteria were selected: 1) all preclinical controlled animal model studies with PC-based approaches for bone and/or cartilage regeneration; 2) data were measured as new bone and/or cartilage formation (%) with the utilization of PCs-based strategies.

The exclusion criteria included: 1) review articles, abstracts, letters, editorials, correspondences, and case reports; 2) PCs that were genetically modified or not isolated from the periosteum.

### Study selection and data collection process

The information retrieved from the database was compiled, and any duplicate entries were removed. The title and abstract were evaluated based on eligibility criteria by the two authors separately. Studies considered ineligible by the two authors were excluded immediately, while studies considered ineligible by one author but eligible by the second author were retained for reading the full text. Researches not excluded were read in full text by two reviewers, who then chose studies that met the eligibility criteria and conducted data extraction. Any disagreements were then resolved through discussion and consensus with all the reviewers.

Data from selected studies were retrieved and gathered in detail in one document. Reports of the following variables were extracted from each study: author(s), year of publication, species, age, sex, animal model, tissue origin, types of tissue regeneration, source of MSCs, defect type, implant site, scaffold, density, scaffold size, treatment duration, measurement, and main findings.

### Quality assessment

The quality assessment in selected studies was evaluated independently by 2 authors based on the risk of bias (RoB) tool of Systematic Review Centre for Laboratory Animal Experimentation (SYRCLE) (Hooijmans et al., 2014). The tool contains 8 criteria designed to determine the appraisal of validity, which was assessed as low, high, or unclear. The following 8 questions were included: 1) Was the allocation sequence adequately generated and applied? 2) Were the groups similar at baseline or adjusted for confounders? 3) Was the allocation adequately concealed? 4) Were the animals randomly housed during the experiment? 5) Were the caregivers and/or investigators adequately blinded? 6) Were animals selected at random during outcome assessment? 7) Was the outcome assessment adequately blinded? 8) Were incomplete outcome data adequately addressed? Furthermore, the other two questions were applied to avoid excessive items being rated as unclear because of poor reporting details of included studies: 1) Was it stated that the experiment was randomized at any level? 2) Was it stated that the experiment was blinded at any level?” (Hutmacher and Sittinger, 2003; Chen et al., 2012). When evaluating the quality of included studies, the quality of question 4 was scored as low if all experimental interventions were present in one animal. In addition, the risk of bias for the sixth question was always considered low if the outcome of control and intervention groups of included studies was assessed at the same time. Any disagreements were then resolved through discussion and consensus with all the reviewers.

## Results

### Study selection

**FIGURE 1:**
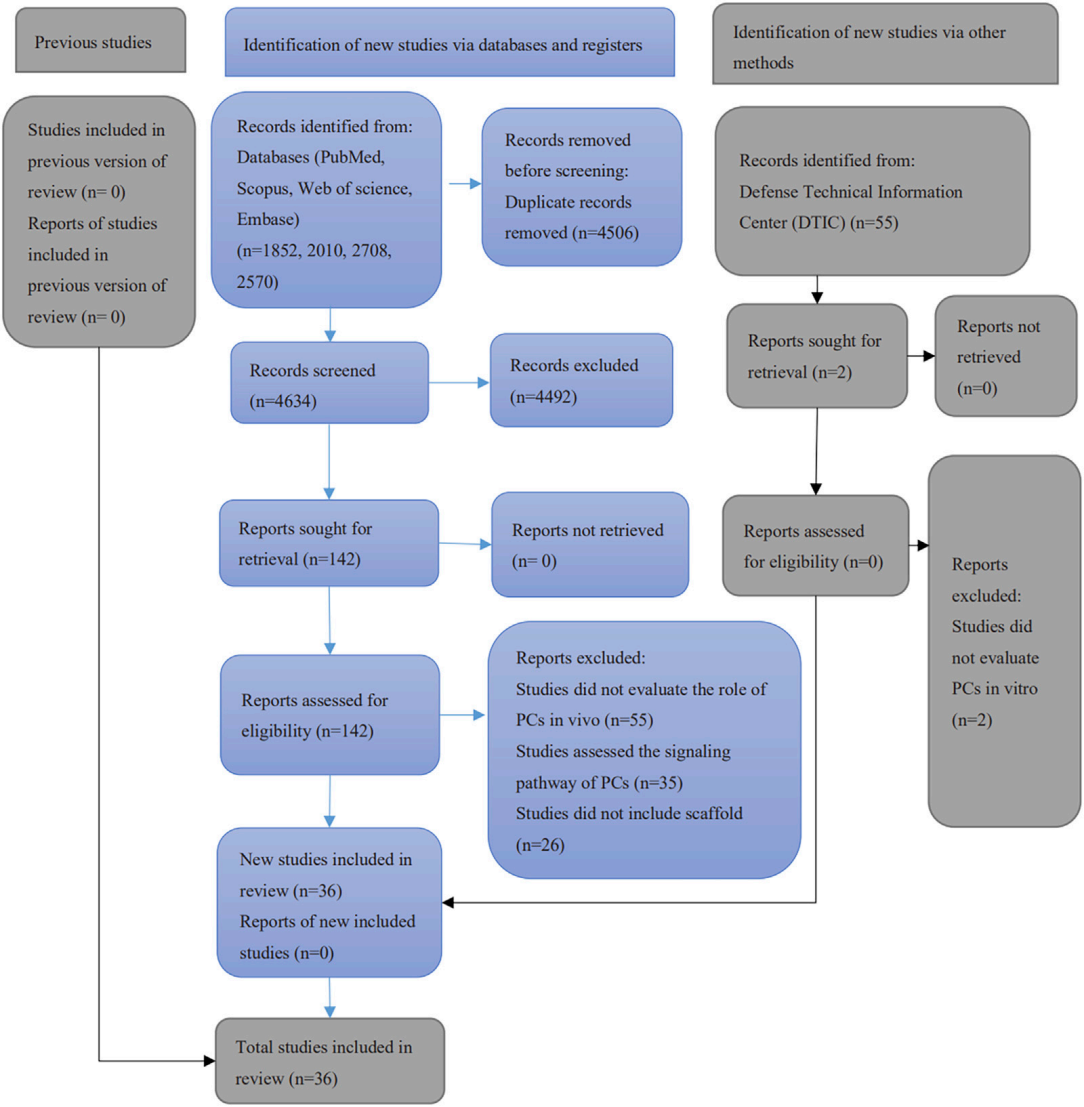
Flow chart of the literature search and results.

A total of 9140 papers were initially retrieved from electronic search, including 1852 articles from PubMed/MEDLINE, 2570 articles from Embase, 2010 articles from Scopus and 2708 from Web of Science. A manual search of the included references yielded a further 5. After removing the duplicates, 4634 publications remained. None of the 55 articles retrieved from the gray literature was considered eligible. Of these, 4492 were excluded after the assessment of abstracts and titles. After the full-text reading, 106 publications were excluded since they did not meet the eligibility criteria. Finally, 36 studies were included in this systematic review (Figure 1). Among them, 30 studies evaluated the potential of PCs in bone formation, 4 studies assessed the PCs in cartilage regeneration and 2 included both in one study.

### Study characteristics

Data from the 36 included publications in bone and cartilage regeneration are presented in Table 2 and Table 3. PCs were harvested from human, rabbit, mouse, sheep, calf, dog and pig. Both male and female samples were included in selected studies. Ages for human samples ranged from 15 years to 83 years, while for animals, ages ranged from 4 weeks to 1.5 years. The femur and tibia were tissue origins used in most studies, while cranium, mandible, radius, and ilium were also included in certain studies (Jaquiéry et al., 2005; Agata et al., 2007; Lee et al., 2011; Matsushima et al., 2011; Lee et al., 2013; Iuchi et al., 2020; Perka et al., 2000; Moreira-Gonzalez et al., 2005; Maréchal et al., 2008; Ribeiro et al., 2010a; Li et al., 2011; Matsushima et al., 2011; Paulo Ade et al., 2011; Leijten et al., 2016). BMSCs were the most widely used MSCs in combination with PCs, and dental pulp stem cells (Annibali et al., 2013), synovial membrane MSCs (De Bari et al., 2008; Li et al., 2011), adipose-derived MSCs (Lee et al., 2011; Li et al., 2011; Stockmann et al., 2012), periodontal ligament cells (Tsumanuma et al., 2011), and muscle membrane MSCs (Li et al., 2011) were also applied. Most researchers chose mouse and rabbit as animal models. The implant site includes subcutaneous pockets, calvaria, mandible, tibia, ulna, ear and femur. Multiple scaffolds were used in selected studies, β-tricalcium phosphate (β-TCP) (Agata et al., 2007; Chen et al., 2011; Chen et al., 2012; Annibali et al., 2013; Chen et al., 2015), 3D collagen (Sakata et al., 2006; Ryu et al., 2011), BioOss (Jaquiéry et al., 2005; Katagiri et al., 2019), Collagraft (Eyckmans and Luyten, 2006; Chang et al., 2012; van Gastel et al., 2012), and Polydioxanone/pluronic F127 (Lee et al., 2011; Lee et al., 2013), and the size of the scaffold also varies. The treatment duration ranged from 4 weeks to 3 months. Histomorphometry was used to measure the outcomes in most studies, and the remaining researchers mainly selected micro-CT and X-rays.

**TABLE 2:** Characteristics and main results of the included studies.

Author(s)	Species	Age	Sex	Tissue origin	Animal model	Tissue regeneration	Source of MSCs	Main findings
Agata et al. (2007)	Human	18–24y	Male and Female	Mandible	Mouse	Bone	PCs, BMSCs	PCs-based>BMSCs-based (*p* < 0.05)
Annibali et al. (2013)	Human	N	N	Teeth	Mouse	Bone	PCs,DPSCs	DPSCs-based, PCs-based<bare scaffold (*p* < 0.05)
Bakker et al. (2008)	Rabbit	23w	Female	Tibia	Rabbit	Bone	PCs	PCs-based<bare scaffold (NS)
De Bari et al. (2008)	Human	24–83y	N	Tibia	Mouse	Bone	PCs, SM-MSCs	PCs-based>SM-MSCs-based (NS)
Chang et al. (2012)	Rabbit	N	N	Tibia	Rabbit	Cartilage	PCs	PCs-based>bare scaffold (*p* < 0.05)
Chen et al. (2011)	Human	22–30y	Male and Female	Limb	Mouse	Bone	PCs, BMSCs	PCs-based>BMSCs-based>bare scaffold (*p* < 0.05)
Chen et al. (2012)	Human	22–30y	Male and Female	Limb	Mouse	Bone	PCs,BMSCs	BMSCs+PCs-based>PCs-based>BMSCs-based>bare scaffold (*p* < 0.05)
Chen et al. (2015)	Human	22–30y	Male and Female	Tibia	Rabbit	Bone	PCs,BMSCs	BMSCs+PCs-based>PCs-based>BMSCs-based>bare scaffold (*p* < 0.05)
Eyckmans and Luyten (2006)	Human and rabbit	Human: 15-26y; Rabbit: 11–34w	Female	Tibia	Mouse	Bone	PCs	Human PCs-based>rabbit PCs-based>bare scaffold (NS)
van Gastel et al. (2012)	Human and mouse	Human:N/A; mouse: 7–9w	Male	Human: tibia; mouse: femur and tibia	Mouse	Bone	PCs	Mouse PCs-based> human PCs-based>bare scaffold (NS)
González-Gil et al. (2019)	Mouse	10–12w	N	Femur	Mouse	Bone	PCs,BMSCs	PCs-based>bare scaffold>BMSCs based (NS)
Iuchi et al. (2020)	Calves	1–6 m	N	Cranium, mandible, radius, and ilium	Mouse	Cartilage	PCs	PCs-based>bare scaffold (NS)
Jaquiéry et al. (2005)	Human	21–80y	Male and Female	Mandible	Mouse	Bone	PCs,BMSCs	PCs-based<BMSC based (*p* < 0.05)
Katagiri et al. (2019)	Mouse	11–13w	Female	Femur and tibia	Mouse	Bone	PCs	PCs-based>bare scaffold (NS)
Knothe Tate et al. (2011)	Sheep	N	N	Femur	Sheep	Bone	PCs	PCs-based>bare scaffold (*p* < 0.05)
Lammens et al. (2020)	Sheep	N	Female	Tibia	Sheep	Bone	PCs	PCs-based>bare scaffold (*p* < 0.05)
Lee et al. (2011)	Human	15–18y	N	Mandible	Pig	Bone	PCs AD-MSCs	PCs+AD-MSCs-based>PCs-based scaffold>bare scaffold (NS)
Lee et al. (2013)	Human	15–18y	N	Buccal fat pad and mandible	Pig	Bone	PCs	PCs-based>bare scaffold (*p* < 0.05)
Leijten et al. (2016)	Human	14.9 ± 2.1y	male and female	N	Mouse	Cartilage	PCs	PCs-based>bare scaffold (*p* < 0.05)
Li et al. (2011)	Rabbit	N	N	Tibia	Rabbit	Cartilage	BMSCs, PCs, SM-MSCs, AD-MSCs, MD-MSCs	BMSCs-based>PCs, SM-MSCs, AD-MSCs, MD-MSCs-based>bare scaffold (*p* < 0.05)
Maréchal et al. (2008)	Rabbit	11w	N	Tibia	Rabbit	Bone	PCs	PCs-based<bare scaffold (NS)
Matsushima et al. (2011)	Calf	1–6 m	N	Cranium, mandible, radius and ilium	Mouse	Bone and Cartilage	PCs	Cranium>ilium>radius>mandible PCs-based>bare scaffold (NS)
Miyamoto et al. (2004)	Rabbit	N	N	Calvaria	Rabbit	Bone	PCs	PCs-based>bare scaffold (*p* < 0.05)
Moreira-Gonzalez et al. (2005)	Rabbit	N	Female	Tibia	Rabbit	Bone	PCs	PCs-based>bare scaffold (*p* < 0.05)
Paulo Ade et al. (2011)	Mouse	N	Male	Frontal-parietal region	Mouse	Bone	PCs	PCs-based<bare scaffold (NS)
Perka et al. (2000)	Rabbit	16w	N	Tibia	Rabbit	Bone	PCs	PCs-based>bare scaffold (*p* < 0.05)
Ribeiro et al. (2010a)	Dog	1.5y	N	Mandible	Dog	Bone	PCs, BMSCs	PCs-based>BMSC-based (NS)
Ribeiro et al. (2010b)	Dog	1.5y	N	Mandible	Dog	Bone	PCs, BMSCs	PC-based>bare- based (*p* < 0.05)
Ryu et al. (2011)	Human	N	N	Mandible	Pig	Bone	PCs	PCs-based>bare scaffold (NS)
Sakata et al. (2006)	Human	22y	Female	Mandible	Mouse	Bone	PCs	PCs-based>bare scaffold (*p* < 0.05)
Stockmann et al. (2012)	Pig	18 m	Female	Tibia	Pig	Bone	PCs, BMSCs, AD-MSCs	PCs-based, AD-MSCs-based, BMSCs-based>bare scaffold (*p* < 0.05)
Tsumanuma et al. (2011)	Dog	N	Male	Mandible	Dog	Bone	PCs, BMSCs, PDLCs	PDLCs-based>PCs-based>BMSCs-based>bare scaffold (NS)
Yin et al. (2018)	Mouse	4w	Male	N	Mouse	Bone	PCs	PCs-based>bare scaffold (*p* < 0.05)
Yoo et al. (2021)	Rabbit	12w	Male	Calvarium and tibia	Rabbit	Bone and Cartilage	PCs	Bone: PCs-based>bare scaffold (NS); Cartilage: PCs-based>bare scaffold (*p* < 0.05)
Zhang et al. (2012)	Rabbit	N	N	Ulna	Rabbit	Bone	PCs, BMSCs	PCs+BMSCs-based>BMSCs-based, PCs-based> bare scaffold (*p* < 0.05)
Zhu et al. (2006)	Dog	N	N	Mandible	Mouse	Bone	PCs, BMSCs, alveolar bone cells	PCs-based>alveolar bone cells-based>BMSCs-based (*p* < 0.05)

AD-MSCs, adipose-derived mesenchymal stem cells; DPSCs, dental pulp stem cells; MD-MSCs, muscle membrane mesenchymal stem cells; PDLCs, periodontal ligament cells; SM-MSCs, synovial membrane mesenchymal stem cells.

**TABLE 3:** Information related to scaffolds used in tissue engineering.

Author(s)	Implant site	Defect type	Scaffold	Density	Scaffold size	Treatment duration	Measurement
Agata et al. (2007)	Subcutaneous pocket	Bone augmentation	β-TCP	1*10(6)	N	4 weeks	Histomorphometry
Annibali et al. (2013)	Calvaria	Monocortical	GDPB/10% porcine collagen; granular β-TCP; Aga/nHA	1*10(6)	A pore size of 50 to 500 um	8 weeks	Histomorphometry
Bakker et al. (2008)	Tibiae	Monocortical	HA; Ti; PH70aTCP	2*10(7)	Hight: 6 mm; Diameter: 20 mm	10 weeks	Histomorphometry
De Bari et al. (2008)	Extra-articular bone	Ectopic	N	5*10(5)	N	8 weeks	Macroscopic, Histomorphometry
Chang et al. (2012)	Subcutaneous pockets	Bone augmentation	Collagraft	N	N	8 weeks	Histomorphometry
Chen et al. (2011)	Subcutaneous pocket	Ectopic	β-TCP	9*10(5)	Hight: 2 mm; Diameter: 6 mm	8 weeks	Histomorphometry
Chen et al. (2012)	Subcutaneous pocket	Ectopic	β-TCP	9*10(5)	Hight: 2 mm; Diameter: 6 mm	8 weeks	Histomorphometry
Chen et al. (2015)	Tibia	Bicortical	3D β-TCP	1.2*10(6)	Hight: 10 mm; Diameter: 6 mm	12 weeks	Histomorphometry
Eyckmans and Luyten (2006)	Subcutaneous pockets	Bone augmentation	Collagraft	5*10(6)	Hight: 3 mm; Diameter: 3 mm	8 weeks	Histomorphometry
van Gastel et al. (2012)	Subcutaneous pockets	Bone augmentation	Collagraft	1*10(6)	3*3*3 mm	8 weeks	Histomorphometry
González-Gil et al. (2019)	Mouse	Bicortical	PCL	6.7*10(4)	2 mm pore size and 6 mm in height	10 weeks	Micro-CT
Iuchi et al. (2020)	Subcutaneous pockets	Bone augmentation	3D scaffold hydroxyapatitepoly L-lactic-3-caprolactone (HA-P[LA/CL])	N	length, 17 mm; width, 7 mm; height, 5 mm	20 weeks	Histomorphometry
Jaquiéry et al. (2005)	Subcutaneous pocket	Ectopic	BioOss; Vitoss	N	Vitoss: 6*6*6 mm	8 weeks	Histomorphometry
Katagiri et al. (2019)	Subcutaneous pocket	Ectopic	BioOss and Copios	1.7*10(6)	Hight: 3–5 mm; Diameter: 3 mm	6 weeks	microCT
Knothe Tate et al. (2011)	Femur	Ectopic	Periosteum substitute and collagen	N	A long sleeve (3.56*10 cm) with four 2 cm wide pockets	16 weeks	Histomorphometric, m-CT
Lammens et al. (2020)	Tibia	Bicortical	Dicalciumphosphate (DCP)	1*10(6)	Diameter: 2 mm Thickness: 4mm; Central hole: 6 mm	16 weeks	Nano CT
Lee et al. (2011)	Mandible	Bicortical	Polydioxanone/pluronic F127	2*10(5)	Hight: 5mm; Diameter:15 mm	12 weeks	X-rays, CT, histomorphometry
Lee et al. (2013)	Mandible	Bicortical	Polydioxanone/pluronic F127 scaffold	2*10(5)	Hight: 5 mm; Diameter: 15 mm	12 weeks	X-rays, CT scans
Leijten et al. (2016)	Subcutaneous pockets	Bone augmentation	Microaggregates, hydrogels of collagen type I	1*10(7)	N	3 weeks	Histomorphometry
Li et al. (2011)	femoral	Monocortical	demineralized bone matrix (DBM)	3.5*10(5)	4 mm diameter and 3 mm thickness	12 weeks	Histomorphometry
Maréchal et al. (2008)	Calvaria	Bone augmentation	TCP; HA/TCP	2*10(7)	TCP: 10*8*6 mmHA/TCP: 5 mm*5 mm*5 mm	12 weeks	Histomorphometry
Matsushima et al. (2011)	Subcutaneous pockets	Bone augmentation	Hydroxyapatite-poly	N	1*1*0.5 cm	20 weeks	X-rays, histology, gene expression levels
Miyamoto et al. (2004)	Calvaria	Bicortical	PLLA +collagen	1*10(6)	Hight: 3.5 mm; Diameter: 4.5 mm	12 weeks	Histomorphometry
Moreira-Gonzalez et al. (2005)	Calvaria	Bicortical	Bioglass	N	N	12 weeks	Histomorphometry, X-rays
Paulo Ade et al. (2011)	Calvaria	Bicortical	HA-COL Scaffold	1*10(5)	Hight: 1.5 mm; Diameter: 8 mm	1/3 month	Histomorphometry
Perka et al. (2000)	Ulna	Ectopic	PLGA	5*10(4)	N	4 weeks	X-rays
Ribeiro et al. (2010a)	Alveolar bone	Monocortical	Collagen sponge	2*10(7)	N	3 month	Histomorphometry
Ribeiro et al. (2010b)	Mandible	Bicortical	BD 3D Scaffold Composite	2*10(7)	N	3 months	Histomorphometry
Ryu et al. (2011)	Mandible	Bicortical	3D collagen scaffold	3*10(4)	N	4 weeks	Histomorphometry
Sakata et al. (2006)	Calvaria	Bicortical	3D collagen scaffolds	5*10(4)	N	5 weeks	X-rays
Stockmann et al. (2012)	Calvaria	Monocortical	Collagen	2*10(6)	N	3 month	Histomorphometry, X-rays
Tsumanuma et al. (2011)	Mandible	Bicortical	PGA sheets	N	N	8 weeks	Histomorphometry
Yin et al. (2018)	Calvaria	Bicortical	chitosan–collagen (CS/COL)	1*10(5)	Diameter: 8 mm	12 weeks	MicroCT, Histomorphometry
Yoo et al. (2021)	Ear	Bone augmentation	N	N	N	6 weeks	Histomorphometry
Zhang et al. (2012)	Ulna	Bicortical	Porous PLGA	N	4 × 15 mm	12 weeks	Histomorphometry, gross observation, X-ray, micro-CT
Zhu et al. (2006)	Subcutaneous pocket	Ectopic	Fibrin glue	1*10(6)	400 mL glue	12 weeks	Histomorphometry

Most of the 32 included studies that evaluated the bone formation capacity of PCs compared the osteogenic effects of scaffolds seeded with PCs to those without implanted cells. 27 publications reported positive results in new bone formation and 13 of them demonstrated significant statistical differences. Conversely, findings from one additional study were inconsistent with the aforementioned observations when using PCs and dental pulp stem cells in tissue engineering (Annibali et al., 2013). Furthermore, 9 studies compared the osteogenic performance of PCs to BMSCs (Jaquiéry et al., 2005; Agata et al., 2007; Ribeiro et al., 2010a; Ribeiro et al., 2010b; Chen et al., 2011; Li et al., 2011; Chen et al., 2012; Chen et al., 2015; González-Gil et al., 2019). Among them, 5 studies indicated that PCs exhibited stronger *in vivo* osteogenic differentiation capabilities compared to BMSCs with statistical differences (Jaquiéry et al., 2005; Agata et al., 2007; Ribeiro et al., 2010b; Chen et al., 2011; Chen et al., 2015). Regarding the cartilage capacity, all 6 studies evaluated the chondrogenic ability of PCs and demonstrated promising results (Li et al., 2011; Matsushima et al., 2011; Chang et al., 2012; Leijten et al., 2016; Iuchi et al., 2020; Yoo et al., 2021). 4 studies reported significant differences in new cartilage regeneration (Li et al., 2011; Chang et al., 2012; Leijten et al., 2016; Yoo et al., 2021). One study (Li et al., 2011) compared the chondrogenic ability of BMSCs and PCs and showed the stronger chondrogenic potential of BMSCs. The chondrogenic ability of synovial membrane MSCs, adipose-derived MSCs and muscle membrane MSCs were also evaluated and demonstrated (Li et al., 2011). Furthermore, another study compared the capacity of bone and cartilage formation in periosteum from different sources (Matsushima et al., 2011). The results showed that cranial and mandibular periosteal tissues increased the bone and cartilage formation capacity most and least prominently, respectively.

### Quality assessment

**FIGURE 2:**
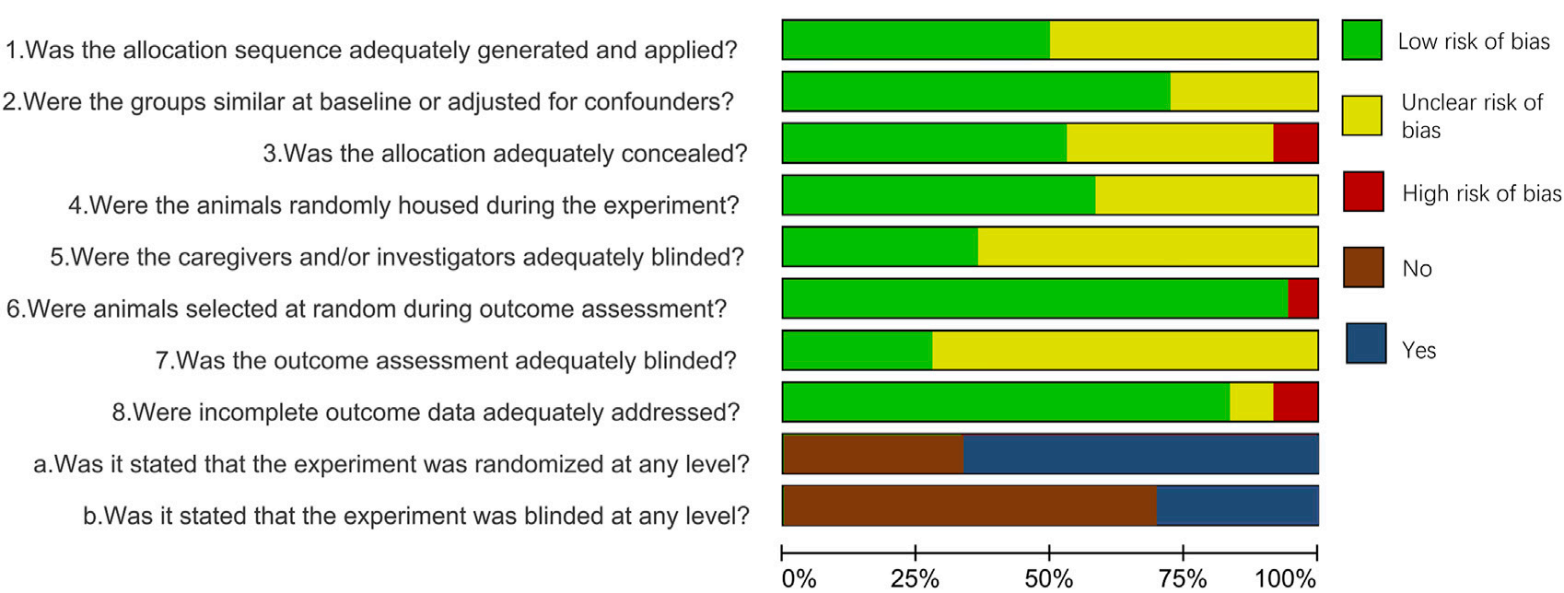
Risk of bias.

Figure 2 and Table 4 summarize the risk of bias in the included studies. Regarding selection bias, 18 studies included the randomization of the experimental process, while the sequence generation of the remaining 18 studies was considered an unclear risk of bias since they did not mention the randomization. Among the 36 included studies, 26 indicated that the baseline characteristics such as age, gender and weight were similar between the experimental and control groups. 19 of the studies were considered a low risk of bias since they mentioned the allocation concealment. However, 3 studies presented a high risk of bias in allocation concealment because the experimenters were aware of which group the samples came from. Furthermore, for performance bias, 21 researches were assessed as low risk in terms of “random housing,” while other the 15 studies had an unclear risk because the authors could not determine if the animals were randomly housed in the experiments. Unclear bias risks in terms of blinding were identified in 23 studies. Regarding detection bias, 34 studies were assessed as low bias risk in “random outcome assessment,” while 2 studies had a high risk of bias because animals were not randomly selected. In the seventh item, 10 of the included studies were considered a low risk of bias because of the use of blinding for outcome assessment. For attrition bias, 30 studies were assessed as low risk, while 3 studies presented a high risk of bias because of the non-use or exclusion of incomplete data. For the two additional questions, 24 studies stated that the experiment was randomized at any level, while only 11 researches indicated that the experiment was blinded at any level.

**TABLE 4:** Quality assessment of included studies.

Author(s)	Question number according to SYRCLE’s risk of bias tool										Overall
	1	2	3	4	5	6	7	8	a	b	
Agata et al. (2007)	low	low	low	low	unclear	low	unclear	unclear	no	no	unclear
Annibali et al. (2013)	low	low	unclear	low	unclear	low	unclear	low	yes	yes	low
Bakker et al. (2008)	low	low	low	low	unclear	high	unclear	low	yes	yes	unclear
De Bari et al. (2008)	unclear	unclear	unclear	unclear	unclear	low	unclear	unclear	no	no	unclear
Chang et al. (2012)	low	low	low	low	low	low	low	low	yes	yes	low
Chen et al. (2011)	unclear	unclear	high	unclear	unclear	low	unclear	low	yes	yes	unclear
Chen et al. (2012)	unclear	unclear	unclear	unclear	unclear	low	unclear	low	yes	no	low
Chen et al. (2015)	unclear	low	unclear	low	unclear	low	unclear	low	yes	no	low
Eyckmans and Luyten (2006)	unclear	low	high	unclear	unclear	low	unclear	low	no	no	unclear
van Gastel et al. (2012)	unclear	unclear	unclear	unclear	unclear	low	unclear	unclear	no	no	low
González-Gil et al. (2019)	unclear	unclear	unclear	unclear	unclear	low	unclear	low	no	no	low
Iuchi et al. (2020)	unclear	low	unclear	unclear	unclear	low	unclear	low	yes	no	unclear
Jaquiéry et al. (2005)	unclear	unclear	unclear	unclear	unclear	low	unclear	low	yes	no	low
Katagiri et al. (2019)	unclear	unclear	high	unclear	unclear	high	unclear	low	no	no	unclear
Knothe Tate et al. (2011)	unclear	low	low	unclear	unclear	low	unclear	high	yes	no	low
Lammens et al. (2020)	low	low	low	low	unclear	low	unclear	low	yes	no	low
Lee et al. (2011)	low	low	low	low	low	low	low	low	yes	yes	low
Lee et al. (2013)	low	low	low	low	low	low	low	low	yes	yes	low
Leijten et al. (2016)	unclear	low	unclear	unclear	unclear	low	unclear	low	no	no	unclear
Li et al. (2011)	low	low	low	low	low	low	unclear	low	yes	no	low
Maréchal et al. (2008)	low	low	low	low	low	low	unclear	low	yes	no	low
Matsushima et al. (2011)	unclear	unclear	unclear	unclear	unclear	low	low	low	no	no	unclear
Miyamoto et al. (2004)	low	low	low	low	low	low	low	low	yes	yes	low
Moreira-Gonzalez et al. (2005)	unclear	low	unclear	unclear	unclear	low	unclear	high	no	no	unclear
Paulo Ade et al. (2011)	unclear	low	low	unclear	unclear	low	unclear	low	yes	no	low
Perka et al. (2000)	unclear	unclear	unclear	low	unclear	low	unclear	low	no	no	low
Ribeiro et al. (2010a)	low	low	low	low	unclear	low	unclear	low	yes	no	low
Ribeiro et al. (2010b)	low	low	low	low	unclear	low	unclear	low	yes	no	low
Ryu et al. (2011)	low	low	low	low	low	low	low	low	yes	yes	low
Sakata et al. (2006)	low	low	low	low	low	low	unclear	low	yes	no	low
Stockmann et al. (2012)	low	low	low	low	low	low	low	high	yes	yes	unclear
Tsumanuma et al. (2011)	low	low	low	low	low	low	low	low	yes	yes	low
Yin et al. (2018)	unclear	low	unclear	unclear	unclear	low	unclear	low	yes	no	low
Yoo et al. (2021)	low	low	low	low	low	low	low	low	yes	yes	low
Zhang et al. (2012)	low	low	low	low	low	low	low	low	no	no	low
Zhu et al. (2006)	unclear	unclear	unclear	low	low	low	unclear	low	no	no	low

Overall, 25 of the included studies presented a low risk of bias, and 11 researches were regarded as an unclear risk of bias, none of the included studies were scored as a high risk in the quality assessment. 19 of the 27 studies which reported positive results in new bone formation present a low risk of bias although the other 8 publications showed an unclear risk of bias. However, half of the studies (3/6) that evaluate the cartilage formation capacity of PCs showed an unclear risk of bias. In addition, 6 studies indicated the greater bone formation capacity of PCs compared to BMSCs with a low risk of bias, and one showed the stronger chondrogenic potential of BMSCs also presents a low risk of bias.

## Discussion

The objective of this study was to summarize the potential of PCs in terms of bone and cartilage regeneration. Despite an exhaustive search, only 36 articles informed the conclusions of our study, most of which focused on the osteogenic capacity of PCs. To our knowledge, this is the first review that focuses on the characteristics and efficacy of these cells in bone and cartilage regeneration in different models.

The isolation and culture of PCs, which is the first step during tissue engineering, plays an essential role in bone and cartilage regeneration. Of the 36 studies included in this systematic review, most of them isolated PCs by peeling or scrapping away the periosteum covering the bone surface, followed by enzymatic digestion of the tissue (Perka et al., 2000; Jaquiéry et al., 2005; Eyckmans and Luyten, 2006; Sakata et al., 2006; Agata et al., 2007; Bakker et al., 2008; Maréchal et al., 2008; Ribeiro et al., 2010a; Ribeiro et al., 2010b; Chen et al., 2011; Knothe Tate et al., 2011; Lee et al., 2011; Li et al., 2011; Paulo Ade et al., 2011; Ryu et al., 2011; Tsumanuma et al., 2011; Chang et al., 2012; Chen et al., 2012; Stockmann et al., 2012; van Gastel et al., 2012; Lee et al., 2013; Chen et al., 2015; Leijten et al., 2016; Yin et al., 2018; González-Gil et al., 2019; Katagiri et al., 2019; Iuchi et al., 2020; Lammens et al., 2020). Another approach that has been used in several selected studies of our review involves placing the bones free of epiphyses, skeletal muscle, and bone marrow to facilitate their migration and proliferation (Miyamoto et al., 2004; Zhu et al., 2006). A recent protocol has proved that isolated PCs display high osteogenic, chondrogenic, and adipogenic differentiation abilities and demonstrated promising potential *in vivo* (Perrin et al., 2021). Despite variations of animal species and isolation approaches, key features of PCs using analysis of cell surface markers are highly comparable in selected studies. PCs have been demonstrated to express canonical MSCs such as CD51, CD29, CD90, Sca1 and CD105 in mice and CD90, CD73, CD105, CD166 and CD146 in humans (Duchamp de Lageneste et al., 2018).

The potential of PCs for bone regeneration was first proposed in the 19th century (Nakahara et al., 1991). PCs as the source of MSCs in humans for bone tissue generation have also been proved in current studies. After conducting a comprehensive systematic review, the authors found most publications reported positive results in new bone formation with a combination of PCs and multiple scaffolds, including β-tricalcium phosphate (β-TCP), 3D collagen, BioOss, Collagraft, and Polydioxanone/pluronic F127 (Jaquiéry et al., 2005; Eyckmans and Luyten, 2006; Sakata et al., 2006; Agata et al., 2007; Chen et al., 2011; Lee et al., 2011; Ryu et al., 2011; Chang et al., 2012; Chen et al., 2012; van Gastel et al., 2012; Annibali et al., 2013; Lee et al., 2013; Chen et al., 2015; Katagiri et al., 2019). Scaffolds with PCs present significantly higher bone regeneration efficacy than bare scaffolds (Perka et al., 2000; Miyamoto et al., 2004; Moreira-Gonzalez et al., 2005; Sakata et al., 2006; Ribeiro et al., 2010b; Chen et al., 2011; Knothe Tate et al., 2011; Li et al., 2011; Chang et al., 2012; Chen et al., 2012; Stockmann et al., 2012; Zhang et al., 2012; Lee et al., 2013; Chen et al., 2015; Leijten et al., 2016; Yin et al., 2018; Lammens et al., 2020). However, certain biocompatible scaffold materials may not be suitable for *in vivo* implantation (Annibali et al., 2013). Moreira-Gonzalez et al. (2005) found that when repairing rabbit cranial bone defects, the sole implantation of 45S5 bioactive glass was unfavorable for defect repair, possibly due to the release of soluble silica from 45S5 bioactive glass into the environment, which influenced cell metabolism. In addition, *in vivo* experiments using β-TCP scaffolds indicated that scaffolds loaded with human PCs exhibited more neoangiogenesis and mature bone formation compared to those loaded with BMSCs (Chen et al., 2011). Studies have revealed that the characteristics of scaffolds may influence the behavior of implanted cells and ultimately impact the regenerative outcomes of bone tissue engineering (Ryu et al., 2011). To achieve cellular bone reconstruction and remodeling on a scaffold material, two key aspects need to be considered. The first one is that the provided cells should possess strong osteogenic ability, be non-immunogenic, and be easily obtained and manipulated. In addition, the scaffold material should exhibit good biocompatibility, strong osteoconductive properties, excellent absorbability, support MSCs attachment, and promote rapid vascularization (Perka et al., 2000).

Another interesting area regarding the osteogenic differentiation capability of PCs of the included studies is the influence of donor’s age and sources. Regarding the potential influence of donor cell age on osteogenic differentiation capability, researchers concluded that as donor age increases, the thickness and cellular structure of the periosteum decrease (Jaquiéry et al., 2005). The osteogenic potential of PCs from different donor sources can vary among different tissues. For example, one of the included studies compared the capacity of bone and cartilage formation in periosteum from different sources (Matsushima et al., 2011). After 20 weeks of the implantation of PCs, the calvarial periosteum exhibited significantly higher expression of the runx2 and BSP, indicating strong osteogenic potential. On the other hand, the mandibular periosteum constructs showed slower development, and overall gene expression levels analyzed were not high. Accordingly, the osteogenic differentiation abilities of PCs to bone defect may be influenced by factors such as the age of donor cells and the donor sources.

In addition to the osteogenic potential of PCs, recently, researchers have focused on studying the potential of PCs to differentiate into cartilage and exploring their ability to repair bone defects. The inner layer of the periosteum contains osteoprogenitor cells, chondrocytes, and other osteogenic precursor cells, which can serve as the main source for chondrocyte production. *In vitro* experiments have shown that different types of induction culture media can promote the differentiation of PCs into osteoblasts, chondrocytes, and adipocytes, indicating the characteristics of mesenchymal stem cells (van Gastel et al., 2012). Chang et al. (2012) prepared functional PCs sheets from the periosteum of the rabbit tibia and transplanted them into the tibial tendon tunnel. Morphological and histological staining after 8 weeks demonstrated enhanced fibrocartilage formation at the tendon-bone interface, increased collagen fibers, and glycosaminoglycan deposition. In the present study, all 6 studies assessing the chondrogenic ability of PCs demonstrated promising results, and 4 of them reported significant differences in new cartilage regeneration. Accordingly, the potential of PCs in cartilage regeneration could be a promising strategy in tissue engineering.

Inducing MSCs to differentiate into cartilage can be achieved through various methods, such as modifying cell-loaded biomaterials with biomimetic elements like proteins or peptides, and performing *in vitro* pretreatment of the implant. Essentially, these approaches aim to create a microenvironment conducive to cartilage formation. Scholars abroad have found that when PCs micro-aggregates are integrated into biomaterials without exogenous growth factors, compared to single-cell-loaded biomaterials, the former exhibits upregulation of cartilage formation genes and improved formation of cartilage tissue *in vivo* (Leijten et al., 2016). Different sources of periosteal tissue may have an impact on cartilage formation. Iuchi et al. isolated PCs from the skull, mandible, radius, and ilium, and combined them with three-dimensional hydroxyapatite-poly(l-lactic acid-co-ε-caprolactone) (HA-P[LA/CL]) scaffolds, which were then implanted into nude mice. PCs from the tibia of the lower leg showed better bone formation and maturation of chondrocytes in the engineered phalanges (Iuchi et al., 2020).

One of the main objectives of the present study was to pay attention to the comparison between the PCs and BMSCs. The results of those studies evaluating the differences between PCs and BMSCs indicated that PCs exhibited stronger *in vivo* osteogenic differentiation capabilities. For example, Chen et al. cultured human PCs and BMSCs and compared their osteogenic differentiation capabilities *in vitro* and *in vivo* (Chen et al., 2011). The results showed that human PCs demonstrated greater mineralization ability than BMSCs, with higher expression levels of osteopontin, BMP-2, and osteocalcin genes. Studies have shown that the periosteum contains more MSCs compared to bone marrow stroma, and PCs express more osteoprogenitor and chondroprogenitor cells than BMSCs (Zhu et al., 2006; van Gastel et al., 2012). However, no significant differences were found in the histomorphometric analysis of new bone formation among the different sources of MSCs in another study (Stockmann et al., 2012). Ribeiro et al. implanted carriers containing autologous PCs and BMSCs into extraction sockets of adult Beagle dogs. Although the PCs group showed a trend towards higher new bone area values, there were no significant differences in the formation of mineralized nodules and expression of bone markers between the two groups (Ribeiro et al., 2010a). Other sources of MSCs, such as dental pulp stem cells, adipose-derived MSCs; periodontal ligament cells and muscle membrane MSCs have been also investigated in certain studies. However, conclusive conclusions cannot be drawn due to the experimental variabilities that existed and the limited available research. In addition, only one study evaluated the chondrogenic ability of BMSCs and PCs (Li et al., 2011). Although the results showed the stronger chondrogenic potential of BMSCs, the limited available research restricts our ability to draw conclusions. Accordingly, further research is needed to elucidate the differences between PCs and BMSCs and determine which MSCs from different tissue sources have the advantages in terms of chondrogenic potential.

The present study has some limitations. First of all, despite an extensive study, only 36 studies were selected in this systematic review, and only 6 included articles evaluated the cartilage regeneration capacity of PCs, which restricted us from drawing conclusions. In addition, because of the dissimilarity in settings, such as animal models and scaffold types, and most importantly, outcome characterization, a meta-analysis was not feasible. Therefore, a systematic narrative synthesis approach was adopted in accordance with the research questions proposed to thematically explore the results. Further clinical trials and experimental studies are required to confirm the results of this study.

## Conclusion

After conducting a comprehensive literature review, the potential role of PCs in bone and cartilage regeneration has been demonstrated in the current literature. PCs demonstrated beneficial to bone regenerative efficacy compared to the bare scaffold with a low risk of most (19/27) studies reported. However, the cartilage formation capacity of BMSCs still needs to be investigated due to the limited researches available and the certain risk of bias. Moreover, PCs exhibited higher osteogenic capabilities compared to BMSCs in combination with various scaffolds *in vivo* with good evidence. However, the comparative benefits between the PCs and other sources of MSCs in cartilage regeneration remain uncertain. Further researches are required to confirm these results and determine the advantages of MSCs from different tissue origins in terms of chondrogenic and osteogenic potential.

## Data Availability

The original contributions presented in the study are included in the article/Supplementary Material, further inquiries can be directed to the corresponding author.

## References

[B1] Agata H. Asahina I. Yamazaki Y. Uchida M. Shinohara Y. Honda M. J. (2007). Effective bone engineering with periosteum-derived cells. J. Dent. Res. 86, 79–83. 10.1177/154405910708600113 17189468

[B2] Annibali S. Cicconetti A. Cristalli M. P. Giordano G. Trisi P. Pilloni A. (2013). A comparative morphometric analysis of biodegradable scaffolds as carriers for dental pulp and periosteal stem cells in a model of bone regeneration. J. Craniofac Surg. 24, 866–871. 10.1097/scs.0b013e31827ca530 23714898

[B3] Arthur A. Gronthos S. (2020). Clinical application of bone marrow mesenchymal stem/stromal cells to repair skeletal tissue. Int. J. Mol. Sci. 21, 9759. 10.3390/ijms21249759 33371306 PMC7767389

[B4] Atala A. Kasper F. K. Mikos A. G. (2012). Engineering complex tissues. Sci. Transl. Med. 4, 160rv12. 10.1126/scitranslmed.3004890 23152327

[B5] Bakker A. D. Schrooten J. van Cleynenbreugel T. Vanlauwe J. Luyten J. Schepers E. (2008). Quantitative screening of engineered implants in a long bone defect model in rabbits. Tissue Eng. Part C Methods 14, 251–260. 10.1089/ten.tec.2008.0022 18781837

[B6] Bolander J. Ji W. Leijten J. Teixeira L. M. Bloemen V. Lambrechts D. (2017). Healing of a large long bone defect through serum-free *in vitro* priming of human periosteum-derived cells. Stem Cell Rep. 8, 758–772. 10.1016/j.stemcr.2017.01.005 PMC5355567 28196691

[B7] Chang C. H. Chen C. H. Liu H. W. Whu S. W. Chen S. H. Tsai C. L. (2012). Bioengineered periosteal progenitor cell sheets to enhance tendon-bone healing in a bone tunnel. Biomed. J. 35, 473–480. 10.4103/2319-4170.104412 23442360

[B8] Charwat S. Gyöngyösi M. Lang I. Graf S. Beran G. Hemetsberger R. (2008). Role of adult bone marrow stem cells in the repair of ischemic myocardium: current state of the art. Exp. Hematol. 36, 672–680. 10.1016/j.exphem.2008.01.005 18358589

[B9] Chen D. Shen H. He Y. Chen Y. Wang Q. Lu J. (2015). Synergetic effects of hBMSCs and hPCs in osteogenic differentiation and their capacity in the repair of critical-sized femoral condyle defects. Mol. Med. Rep. 11, 1111–1119. 10.3892/mmr.2014.2883 25373389

[B10] Chen D. Shen H. Shao J. Jiang Y. Lu J. He Y. (2011). Superior mineralization and neovascularization capacity of adult human metaphyseal periosteum-derived cells for skeletal tissue engineering applications. Int. J. Mol. Med. 27, 707–713. 10.3892/ijmm.2011.634 21369695

[B11] Chen D. Zhang X. He Y. Lu J. Shen H. Jiang Y. (2012). Co-culturing mesenchymal stem cells from bone marrow and periosteum enhances osteogenesis and neovascularization of tissue-engineered bone. J. Tissue Eng. Regen. Med. 6, 822–832. 10.1002/term.489 22072318

[B12] De Bari C. Dell'Accio F. Karystinou A. Guillot P. V. Fisk N. M. Jones E. A. (2008). A biomarker-based mathematical model to predict bone-forming potency of human synovial and periosteal mesenchymal stem cells. Arthritis Rheum. 58, 240–250. 10.1002/art.23143 18163504

[B13] Dimitriou R. Mataliotakis G. I. Angoules A. G. Kanakaris N. K. Giannoudis P. V. (2011). Complications following autologous bone graft harvesting from the iliac crest and using the RIA: a systematic review. Injury 42, S3–S15. S3–S15. 10.1016/j.injury.2011.06.015 21704997

[B14] Duchamp de Lageneste O. Julien A. Abou-Khalil R. Frangi G. Carvalho C. Cagnard N. (2018). Periosteum contains skeletal stem cells with high bone regenerative potential controlled by Periostin. Nat. Commun. 9, 773. 10.1038/s41467-018-03124-z 29472541 PMC5823889

[B15] Eyckmans J. Luyten F. P. (2006). Species specificity of ectopic bone formation using periosteum-derived mesenchymal progenitor cells. Tissue Eng. 12, 2203–2213. 10.1089/ten.2006.12.2203 16968161

[B16] Goldberg A. Mitchell K. Soans J. Kim L. Zaidi R. (2017). The use of mesenchymal stem cells for cartilage repair and regeneration: a systematic review. J. Orthop. Surg. Res. 12, 39. 10.1186/s13018-017-0534-y 28279182 PMC5345159

[B17] González-Gil A. B. Lamo-Espinosa J. M. Muiños-López E. Ripalda-Cemboráin P. Abizanda G. Valdés-Fernández J. (2019). Periosteum-derived mesenchymal progenitor cells in engineered implants promote fracture healing in a critical-size defect rat model. J. Tissue Eng. Regen. Med. 13, 742–752. 10.1002/term.2821 30785671

[B18] Grayson W. L. Bunnell B. A. Martin E. Frazier T. Hung B. P. Gimble J. M. (2015). Stromal cells and stem cells in clinical bone regeneration. Nat. Rev. Endocrinol. 11, 140–150. 10.1038/nrendo.2014.234 25560703 PMC4338988

[B19] Hooijmans C. R. Rovers M. M. de Vries R. B. Leenaars M. Ritskes-Hoitinga M. Langendam M. W. (2014). SYRCLE's risk of bias tool for animal studies. BMC Med. Res. Methodol. 14, 43. 10.1186/1471-2288-14-43 24667063 PMC4230647

[B20] Hutmacher D. W. Sittinger M. (2003). Periosteal cells in bone tissue engineering. Tissue Eng. 9, S45–S64. 10.1089/10763270360696978 14511470

[B21] Iuchi T. Kusuhara H. Ueda Y. Morotomi T. Isogai N. (2020). Influence of periosteum location on the bone and cartilage in tissue-engineered phalanx. J. Hand Surg. Am. 45, 62.e1–62.e10. 10.1016/j.jhsa.2019.02.002 30902355

[B22] Jaquiéry C. Schaeren S. Farhadi J. Mainil-Varlet P. Kunz C. Zeilhofer H. F. (2005). *In vitro* osteogenic differentiation and *in vivo* bone-forming capacity of human isogenic jaw periosteal cells and bone marrow stromal cells. Ann. Surg. 242, 859–868. 10.1097/01.sla.0000189572.02554.2c 16327496 PMC1409890

[B23] Jukes J. M. van Blitterswijk C. A. de Boer J. (2010). Skeletal tissue engineering using embryonic stem cells. J. Tissue Eng. Regen. Med. 4, 165–180. 10.1002/term.234 19967745

[B24] Katagiri H. Mendes L. F. Luyten F. P. (2019). Reduction of BMP6-induced bone formation by calcium phosphate in wild-type compared with nude mice. J. Tissue Eng. Regen. Med. 13, 846–856. 10.1002/term.2837 30815997

[B25] Knothe Tate M. L. Chang H. Moore S. R. Knothe U. R. (2011). Surgical membranes as directional delivery devices to generate tissue: testing in an ovine critical sized defect model. PLoS One 6, e28702. 10.1371/journal.pone.0028702 22174873 PMC3236208

[B26] Kolios G. Moodley Y. (2013). Introduction to stem cells and regenerative medicine. Respiration 85, 3–10. 10.1159/000345615 23257690

[B27] Lammens J. Maréchal M. Delport H. Geris L. Oppermann H. Vukicevic S. (2020). A cell-based combination product for the repair of large bone defects. Bone 138, 115511. 10.1016/j.bone.2020.115511 32599225

[B28] Lee J. H. Kim J. H. Oh S. H. Kim S. J. Hah Y. S. Park B. W. (2011). Tissue-engineered bone formation using periosteal-derived cells and polydioxanone/pluronic F127 scaffold with pre-seeded adipose tissue-derived CD146 positive endothelial-like cells. Biomaterials 32, 5033–5045. 10.1016/j.biomaterials.2011.03.081 21543114

[B29] Lee J. H. Kim S. W. Kim U. K. Oh S. H. June-Kim S. Park B. W. (2013). Generation of osteogenic construct using periosteal-derived osteoblasts and polydioxanone/pluronic F127 scaffold with periosteal-derived CD146 positive endothelial-like cells. J. Biomed. Mater Res. A 101, 942–953. 10.1002/jbm.a.34393 22961670

[B30] Leijten J. Teixeira L. S. Bolander J. Ji W. Vanspauwen B. Lammertyn J. (2016). Bioinspired seeding of biomaterials using three dimensional microtissues induces chondrogenic stem cell differentiation and cartilage formation under growth factor free conditions. Sci. Rep. 6, 36011. 10.1038/srep36011 27808102 PMC5093556

[B31] Li Q. Tang J. Wang R. Bei C. Xin L. Zeng Y. (2011). Comparing the chondrogenic potential *in vivo* of autogeneic mesenchymal stem cells derived from different tissues. Artif. Cells Blood Substit. Immobil. Biotechnol. 39, 31–38. 10.3109/10731191003776769 21117872

[B32] Li Z. Kupcsik L. Yao S. J. Alini M. Stoddart M. J. (2009). Chondrogenesis of human bone marrow mesenchymal stem cells in fibrin-polyurethane composites. Tissue Eng. Part A 15, 1729–1737. 10.1089/ten.tea.2008.0247 19115827

[B33] Maia Ferreira Alencar C. H. Sampaio Silveira C. R. Cavalcante M. M. Maia Vieira C. G. Diógenes Teixeira M. J. Neto F. A. (2020). Periosteum: an imaging review. Eur. J. Radiol. Open 7, 100249. 10.1016/j.ejro.2020.100249 32923528 PMC7475123

[B34] Maréchal M. Eyckmans J. Schrooten J. Schepers E. Luyten F. P. van Steenberghe D. (2008). Bone augmentation with autologous periosteal cells and two different calcium phosphate scaffolds under an occlusive titanium barrier: an experimental study in rabbits. J. Periodontol. 79, 896–904. 10.1902/jop.2008.070043 18454669

[B35] Matsushima S. Isogai N. Jacquet R. Lowder E. Tokui T. Landis W. J. (2011). The nature and role of periosteum in bone and cartilage regeneration. Cells Tissues Organs 194, 320–325. 10.1159/000324642 21597269 PMC3178095

[B36] Mendes L. F. Katagiri H. Tam W. L. Chai Y. C. Geris L. Roberts S. J. (2018). Advancing osteochondral tissue engineering: bone morphogenetic protein, transforming growth factor, and fibroblast growth factor signaling drive ordered differentiation of periosteal cells resulting in stable cartilage and bone formation *in vivo* . Stem Cell Res. Ther. 9, 42. 10.1186/s13287-018-0787-3 29467016 PMC5822604

[B37] Miyamoto I. Tsuboi Y. Takahashi K. Hyon S. H. Iizuka T. (2004). Enhancement of bone volume in guided bone augmentation by cell transplants derived from periosteum: an experimental study in rabbit calvarium bone. Clin. Oral Implants Res. 15, 308–314. 10.1111/j.1600-0501.2004.01011.x 15142093

[B38] Moher D. Shamseer L. Clarke M. Ghersi D. Liberati A. Petticrew M. (2015). Preferred reporting items for systematic review and meta-analysis protocols (PRISMA-P) 2015 statement. Syst. Rev. 4, 1. 10.1186/2046-4053-4-1 25554246 PMC4320440

[B39] Moreira-Gonzalez A. Lobocki C. Barakat K. Andrus L. Bradford M. Gilsdorf M. (2005). Evaluation of 45S5 bioactive glass combined as a bone substitute in the reconstruction of critical size calvarial defects in rabbits. J. Craniofac Surg. 16, 63–70. 10.1097/00001665-200501000-00013 15699647

[B40] Nakahara H. Goldberg V. M. Caplan A. I. (1991). Culture-expanded human periosteal-derived cells exhibit osteochondral potential *in vivo* . J. Orthop. R. 9 10.1002/jor.1100090402 2045973

[B41] Paulo Ade O. Castro-Silva II Oliveira D. F. Machado M. E. Bonetti-Filho I. Granjeiro J. M. (2011). Repair of critical-size defects with autogenous periosteum-derived cells combined with bovine anorganic apatite/collagen: an experimental study in rat calvaria. Braz Dent. J. 22, 322–328. 10.1590/s0103-64402011000400011 21861033

[B42] Perka C. Schultz O. Spitzer R. S. Lindenhayn K. Burmester G. R. Sittinger M. (2000). Segmental bone repair by tissue-engineered periosteal cell transplants with bioresorbable fleece and fibrin scaffolds in rabbits. Biomaterials 21, 1145–1153. 10.1016/s0142-9612(99)00280-x 10817267

[B43] Perrin S. Julien A. de Lageneste O. D. Abou-Khalil R. Colnot C. (2021). Mouse periosteal cell culture, *in vitro* differentiation, and *in vivo* transplantationin tibial fractures. Bio Protoc. 11, e4107. 10.21769/BioProtoc.4107 PMC8376579 34458401

[B44] Ribeiro F. V. Suaid F. F. Ruiz K. G. Rodrigues T. L. Carvalho M. D. Nociti F. H. (2010b). Peri-implant reconstruction using autologous periosteum-derived cells and guided bone regeneration. J. Clin. Periodontol. 37, 1128–1136. 10.1111/j.1600-051x.2010.01635.x 20969610

[B45] Ribeiro F. V. Suaid F. F. Ruiz K. G. Salmon C. R. Paparotto T. Nociti F. H. Jr (2010a). Periosteum-derived cells as an alternative to bone marrow cells for bone tissue engineering around dental implants. A histomorphometric study in beagle dogs. J. Periodontol. 81, 907–916. 10.1902/jop.2010.090604 20450354

[B46] Roseti L. Parisi V. Petretta M. Cavallo C. Desando G. Bartolotti I. (2017). Scaffolds for bone tissue engineering: state of the art and new perspectives. Mater Sci. Eng. C Mater Biol. Appl. 78, 1246–1262. 10.1016/j.msec.2017.05.017 28575964

[B47] Ryu Y. M. Hah Y. S. Park B. W. Kim D. R. Roh G. S. Kim J. R. (2011). Osteogenic differentiation of human periosteal-derived cells in a three-dimensional collagen scaffold. Mol. Biol. Rep. 38, 2887–2894. 10.1007/s11033-010-9950-3 20107909

[B48] Sakata Y. Ueno T. Kagawa T. Kanou M. Fujii T. Yamachika E. (2006). Osteogenic potential of cultured human periosteum-derived cells - a pilot study of human cell transplantation into a rat calvarial defect model. J. Craniomaxillofac Surg. 34, 461–465. 10.1016/j.jcms.2006.07.861 17157522

[B49] Schardt C. Adams M. B. Owens T. Keitz S. Fontelo P. (2007). Utilization of the PICO framework to improve searching PubMed for clinical questions. BMC Med. Inf. Decis. Mak. 7, 16. 10.1186/1472-6947-7-16 PMC1904193 17573961

[B50] Steinwachs M. R. Guggi T. Kreuz P. C. (2008). Marrow stimulation techniques. Injury 39, S26–S31. 10.1016/j.injury.2008.01.042 18313469

[B51] Stockmann P. Park J. von Wilmowsky C. Nkenke E. Felszeghy E. Dehner J. F. (2012). Guided bone regeneration in pig calvarial bone defects using autologous mesenchymal stem/progenitor cells - a comparison of different tissue sources. J. Craniomaxillofac Surg. 40, 310–320. 10.1016/j.jcms.2011.05.004 21723141

[B52] Su P. Tian Y. Yang C. Ma X. Wang X. Pei J. (2018). Mesenchymal stem cell migration during bone formation and bone diseases therapy. Int. J. Mol. Sci. 19, 2343. 10.3390/ijms19082343 30096908 PMC6121650

[B53] Sun K. Zhou Z. Ju X. Zhou Y. Lan J. Chen D. (2016). Combined transplantation of mesenchymal stem cells and endothelial progenitor cells for tissue engineering: a systematic review and meta-analysis. Stem Cell Res. Ther. 7, 151. 10.1186/s13287-016-0390-4 27724974 PMC5057480

[B54] Tamaddon M. Wang L. Liu Z. Liu C. (2018). Osteochondral tissue repair in osteoarthritic joints: clinical challenges and opportunities in tissue engineering. Biodes Manuf. 1, 101–114. 10.1007/s42242-018-0015-0 30533248 PMC6267278

[B55] Toros T. Ozaksar K. (2021). Reconstruction of traumatic tubular bone defects using vascularized fibular graft. Injury 52, 2926–2934. 10.1016/j.injury.2019.08.013 31455503

[B56] Tsumanuma Y. Iwata T. Washio K. Yoshida T. Yamada A. Takagi R. (2011). Comparison of different tissue-derived stem cell sheets for periodontal regeneration in a canine 1-wall defect model. Biomaterials 32, 5819–5825. 10.1016/j.biomaterials.2011.04.071 21605900

[B57] van Gastel N. Torrekens S. Roberts S. J. Moermans K. Schrooten J. Carmeliet P. (2012). Engineering vascularized bone: osteogenic and proangiogenic potential of murine periosteal cells. Stem Cells 30, 2460–2471. 10.1002/stem.1210 22911908

[B60] Yin J. Qiu S. Shi B. Xu X. Zhao Y. Gao J. (2018). Controlled release of FGF-2 and BMP-2 in tissue engineered periosteum promotes bone repair in rats. Biomed. Mater 13, 025001. 10.1088/1748-605x/aa93c0 29313523

[B61] Yoo H. Yoon T. Bae H. S. Kang M. S. Kim B. J. (2021). Does periosteum promote chondrogenesis? A comparison of free periosteal and perichondrial grafts in the regeneration of ear cartilage. Arch. Craniofac Surg. 22, 260–267. 10.7181/acfs.2021.00423 34732038 PMC8568495

[B62] Zhang X. Qi Y. Y. Zhao T. F. Li D. Dai X. S. Niu L. (2012). Reconstruction of segmental bone defects in the rabbit ulna using periosteum encapsulated mesenchymal stem cells-loaded poly (lactic-co-glycolic acid) scaffolds. Chin. Med. J. Engl. 125, 4031–4036. 10.3760/cma.j.issn.0366-6999.2012.22.022 23158138

[B63] Zhu J. Xiong J. Ji W. (2023). A systematic review of bone marrow stromal cells and periosteum-derived cells for bone regeneration. Tissue Eng. Part B Rev. 29, 103–122. 10.1089/ten.teb.2022.0115 36066333

[B64] Zhu S. J. Choi B. H. Huh J. Y. Jung J. H. Kim B. Y. Lee S. H. (2006). A comparative qualitative histological analysis of tissue-engineered bone using bone marrow mesenchymal stem cells, alveolar bone cells, and periosteal cells. Oral Surg. Oral Med. Oral Pathol. Oral Radiol. Endod. 101, 164–169. 10.1016/j.tripleo.2005.04.006 16448916

